# A Quantitative Comparison of the Behavior of Human Ventricular Cardiac Electrophysiology Models in Tissue

**DOI:** 10.1371/journal.pone.0084401

**Published:** 2014-01-08

**Authors:** Mohamed M. Elshrif, Elizabeth M. Cherry

**Affiliations:** 1 B. Thomas Golisano College of Computing and Information Sciences, Rochester Institute of Technology, Rochester, New York, United States of America; 2 School of Mathematical Sciences, Rochester Institute of Technology, Rochester, New York, United States of America; Georgia State University, United States of America

## Abstract

Numerical integration of mathematical models of heart cell electrophysiology provides an important computational tool for studying cardiac arrhythmias, but the abundance of available models complicates selecting an appropriate model. We study the behavior of two recently published models of human ventricular action potentials, the Grandi-Pasqualini-Bers (GPB) and the O'Hara-Virág-Varró-Rudy (OVVR) models, and compare the results with four previously published models and with available experimental and clinical data. We find the shapes and durations of action potentials and calcium transients differ between the GPB and OVVR models, as do the magnitudes and rate-dependent properties of transmembrane currents and the calcium transient. Differences also occur in the steady-state and S1–S2 action potential duration and conduction velocity restitution curves, including a maximum conduction velocity for the OVVR model roughly half that of the GPB model and well below clinical values. Between single cells and tissue, both models exhibit differences in properties, including maximum upstroke velocity, action potential amplitude, and minimum diastolic interval. Compared to experimental data, action potential durations for the GPB and OVVR models agree fairly well (although OVVR epicardial action potentials are shorter), but maximum slopes of steady-state restitution curves are smaller. Although studies show alternans in normal hearts, it occurs only in the OVVR model, and only for a narrow range of cycle lengths. We find initiated spiral waves do not progress to sustained breakup for either model. The dominant spiral wave period of the GPB model falls within clinically relevant values for ventricular tachycardia (VT), but for the OVVR model, the dominant period is longer than periods associated with VT. Our results should facilitate choosing a model to match properties of interest in human cardiac tissue and to replicate arrhythmia behavior more closely. Furthermore, by indicating areas where existing models disagree, our findings suggest avenues for further experimental work.

## Introduction

Over the last several decades, mathematical models of the electrophysiology of cardiac cells have become an important resource for studying the mechanisms underlying cardiac arrhythmias. These models generally use systems of coupled ordinary differential equations to describe the movement of sodium calcium, and potassium ions across the cell membrane through different ion channels and the changes in membrane potential during an action potential. Many models also represent subcellular processes, such as cycling in intracellular calcium that is responsible for contraction at the cellular level. Tissue-level phenomena can be studied by including cell-to-cell coupling, normally through a diffusion term, to allow for propagation of electrical waves. The models can be used to study the normal electrical state of the heart, in which electrical waves remain coherent to stimulate a coordinated contraction, and arrhythmic states, in which electrical wave disturbances, such as reentry and fractionation, compromise the heart's ability to pump blood.

The advantages models provide, including reproducibility, the ability to vary parameters systematically, and ready access to all simulation results at high spatial and temporal resolution, serve as a useful complement to traditional biological experiments and can be used to develop and to perform preliminary tests of hypotheses. As modeling cardiac electrophysiology has become a more important investigative tool, models have grown in number, specificity, and complexity [1], [2]. For example, models have been developed to describe different regions of the heart, including atria, ventricles, sinoatrial node, atrioventricular node, and Purkinje network, and in many cases are designed to reproduce the behavior of cells of particular species, sometimes under various disease conditions. Models may be developed to incorporate new experimental data on ion channels or other intracellular processes, to address limitations of previous models, or to represent a specific system or mechanism not previously modeled.

The availability of a large number of mathematical models of cardiac cells leads to challenges in selecting an appropriate model. Even when a particular species and region of the heart are identified, it is often the case that several models are available [3]–[9]. The choice of model becomes especially challenging for simulations in tissue, where electronic coupling can cause the emergent properties in higher spatial dimensions to differ from the characteristics of isolated cells [10], [11]. For example, important properties of alternans in tissue, such as alternans magnitude, range of cycle lengths exhibiting alternans, and even whether alternans occurs at all, can differ significantly from what is observed in single cells [6]. In addition, some tissue behavior, such as reentrant wave dynamics, currently cannot be predicted from the properties of isolated cells.

Models of human cells and tissue are of particular importance because of their potential for clinical relevance. A number of mathematical descriptions of human ventricular cells have been published over the past decade and a half [12]–[17]. More recently, two new models were developed: the Grandi-Pasqualini-Bers (GPB) model [18] and the O'Hara-Virág-Varró-Rudy (OVVR) model [19]. These models incorporated more detailed physiological data concerning intracellular calcium dynamics and transmembrane currents. Although the authors established model behavior in isolated cells and for some conditions in tissue, important dynamical properties, including the behavior of reentrant waves, have not been shown previously. In this manuscript, we analyze quantitatively the GPB and OVVR models with an emphasis on rate-dependent properties associated with tachyarrhythmias and compare the dynamics of these models with each other and, where appropriate, with other models of human ventricular cells [12], [14]–[17].

## Methods

### Electrophysiological model formulations

In this study, we focus on two recently published models of human ventricular cells derived from different experimental data: the Grandi-Pasqualini-Bers (GPB) model [18] and the O'Hara-Virág-Varró-Rudy (OVVR) model [19]. The models have different formulations: the GPB model uses 38 state variables and 14 transmembrane currents, whereas the OVVR model has 41 state variables and 12 transmembrane currents. Eleven currents are common to both models: the fast Na^+^ (I_Na_), L-type Ca^2+^ (I_CaL_), transient outward K^+^ (I_to_), rapidly and slowly activating delayed rectifier K^+^ (I_Kr_ and I_Ks_, respectively), inward rectifier K^+^ (I_K1_), Na^+^/Ca^2+^ exchanger (I_NaCa_), Na^+^/K^+^ pump (I_NaK_), sarcolemmal Ca^2+^ pump (I_pCa_), background Na^+^ (I_bNa_), and background Ca^2+^ (I_bCa_) currents. In addition to these, the GPB model includes Ca^2+^-activated Cl^−^ (I_CaCl_ or I_to2_), plateau K^+^ (I_Kp_), and background Cl^−^ (I_bCl_) currents, as well as fast and slow components of I_to_, and the OVVR model includes a background K^+^ current (I_bK_). Another difference in transmembrane currents is the inclusion of both Na^+^ and K^+^ transport through L-type Ca^2+^ channels in the OVVR model, whereas the GPB model accounts for Na^+^ but not K^+^ transport.

Along with differences in transmembrane currents, the two models incorporate different local subspaces, each of which includes ion concentrations and state variables. The GPB model tracks separate Na^+^ and Ca^2+^ concentrations in the junctional cleft (submembrane space near the T-tubules), subsarcolemma (SL) (the submembrane space not near the T-tubules), and cytosol along with the concentration of Ca^2+^ in the sarcoplasmic reticulum (SR) and a single intracellular K^+^ concentration. The OVVR model includes separate cytosol and junctional cleft concentrations of Na^+^, K^+^, and Ca^2+^, along with junctional and network SR Ca^2+^ concentrations.

The different concentrations allow ion channels to sense different ion concentrations depending on their location and thus are related to the spatial distribution of ion channels within the cell. For most ion channels, the GPB model placed 89% of the channels in the SL and the remaining 11% in the junctional cleft, which represents a uniform distribution based on locating 11% of the membrane in the junctional cleft. Although the distribution of channels is uniform, different concentrations of Ca^2+^ and Na^+^ ions in the junctional cleft and SL regions lead to differences in the currents through the channels in those locations. Only I_CaL_ is distributed differently, with 10% of channels in the SL and 90% in the junctional cleft (in close proximity to the T-tubules and RyR Ca^2+^ release channels). Those currents that involve neither Ca^2+^ nor Na^+^ concentrations (I_to_, I_Kr_, I_K1_, I_Kp_, and I_bCl_) are distributed uniformly and do not require any special consideration. For the OVVR model, uniform channel distribution is assumed except for I_CaL_, which is located entirely in the junctional cleft, and I_NaCa_, which is distributed as 20% in the junctional cleft and 80% elsewhere in the membrane, where cytosolic ion concentrations are sensed.

Several other features of the models are noteworthy. The GPB model includes extensive buffering of Ca^2+^ and Na^+^ to regulate ion homeostasis. The OVVR model includes phosphorylation by Ca^2+^/calmodulin-dependent protein kinase II, which affects intracellular Ca^2+^ cycling. In addition, both models include modifications for representing transmural differences in electrophysiological properties. The GPB model includes epicardial and endocardial formulations, which are obtained by varying the maximum conductance of I_to_ alone. In contrast, the OVVR model reproduces epicardial, midmyocardial, and endocardial cells by modifying a large number of parameter values, including maximal conductances of most transmembrane currents and some parameters governing calcium fluxes, along with the inactivation time constants for I_to_.

For comparison, we also present results using the Priebe-Beuckelmann (PB) [12], Iyer-Mazhari-Winslow (IMW) [14], Ten Tusscher-Panfilov (TP) [16], and Bueno-Orovio-Cherry-Fenton (BCF) [17] human ventricular models. The PB, IMW, and TP models have 22, 67, and 19 state variables, respectively, and 10, 13, and 12 transmembrane currents, respectively. The BCF model uses a different formulation focused on reproducing mesoscale electrophysiological properties (such as action potential shape and rate-dependent behavior). It includes 4 state variables and tracks the sum of fast inward, slow inward, and slow outward transmembrane currents. The GPB, OVVR, PB, and TP models rely primarily on Hodgkin-Huxley representations of transmembrane currents, in contrast to the IMW model, which uses Markov formulations (leading to a significantly larger number of state variables). In addition, the IMW model utilizes data obtained from both recombinant human channels and isolated human ventricular epicardial myocytes, whereas the other models are based on isolated human ventricular myocyte data. To represent transmural heterogeneity, the TP and BCF models include formulations for epicardial, endocardial, and midmyocardial cells (through changing the maximum conductances of I_to_ and I_Ks_ for the TP model), whereas the PB and IMW models include only epicardial formulations.

### Measurement of restitution curves

We used both steady-state and S1–S2 restitution protocols to obtain action potential duration (APD) restitution curves for the models in single cells (0D) and in one-dimensional (1D) cables. Action potentials were elicited using a current strength twice diastolic threshold at a cycle length (CL) of 1000 ms; in the 1D case, the stimulus was applied only to one end of the cable. For each protocol, the APD was measured using the voltage threshold corresponding to 90% repolarization after pacing for 30 s at a CL of 1000 ms. This threshold was used to determine the times at which each action potential began and ended throughout the simulation, with linear interpolation used to obtain more accurate times. APD measurements in 1D were taken from the middle of the cable to minimize the effects of current stimulation and the boundaries [20].

For the steady-state protocol, the cell or tissue was paced for 30 s for a range of CLs starting at 1000 ms and decreasing monotonically until 2∶1 block was reached. At each CL, the last APD and the preceding diastolic interval (DI) pair were recorded (if alternans was present, the last two DI, APD pairs were recorded). For the S1–S2 protocol, the cell or tissue was paced for 30 s for a single CL (the S1 CL), after which a second stimulus (the S2 stimulus) was applied after a variable DI. The last DI and APD were recorded, and the process was repeated for a broad range of DIs. The resulting DI, APD pairs were used to construct a single S1–S2 restitution curve corresponding to a particular value of the S1 CL. Because the curve obtained depended on the S1 CL chosen as well, we produced S1–S2 restitution curves for several values of the S1 CL. When plotted together, a series of S1–S2 restitution curves obtained in this way can be used to provide an assessment of memory in APD. In particular, we calculate the memory amplitude, which is the difference in APD between the longest S1 CL used (here, 1000 ms) and the shortest S1 CL before conduction block or alternans for the longest DI tested [6].

Note that it was not always possible for the models to reach a stable steady state, even when assigning a charge carrier to the stimulus current and axial current in tissue [21]. The difference between consecutive APDs after pacing single cells using the GPB and OVVR models at a constant CL for 5 minutes was on the order of 10^−4^ to 10^−3^ ms, as indicated in Table 1. Although the difference between successive APDs was decreasing, it did not appear to saturate.

**Table 1:** Variation in single-cell APD for epicardial formulations of the GPB and OVVR models.

Model	CL (ms)	1000 (ms)	750 (ms)	500 (ms)	400 (ms)
GPB	APD_1_ - APD_n_	−10.02	4.37	3.18	2.91
	APD_n−1_ - APD_n_	−2.20×10^−3^	0.45×10^−3^	0.18×10^−3^	0.08×10^−3^
OVVR	APD_1_ - APD_n_	−10.32	1.63	1.16	0.96
	APD_n−1_ - APD_n_	−2.80×10^−3^	0.56×10^−3^	0.37×10^−3^	0.10×10^−3^

APD_1_ is the first APD measured and APD_n_ is the last APD measured for a given CL after pacing at a fixed cycle length (CL) for 5 minutes. Differences are given in ms. Initial conditions in all cases are as specified in the original model publications.

We used the same steady-state and S1–S2 protocols to measure conduction velocity (CV) restitution curves in 1D cables. The CV was measured between two adjacent cells in the middle of the cable to minimize boundary effects. The resulting CV values were plotted as a function of the preceding DIs recorded in each restitution protocol. S1–S2 CV restitution curves obtained using different S1 CL values were used to assess the presence of memory in CV [17].

### Spiral wave dynamics and tip trajectories

Spiral waves were initiated in two-dimensional (2D) isotropic and homogeneous tissue sheets using a cross-field stimulation protocol [22]. The spiral tip trajectory was tracked using the zero-normal-velocity method [23] by detecting spiral tips as the intersections of the isopotential line V = −60 mV and the line dV/dt  =  0 for both models except for the OVVR midmyocardial cell type, for which we used the isopotential line V = −65 mV. Histograms of APD, DI, and CL values measured to the nearest 1 ms were developed from all points in the sheet during the simulation and were used to calculate the dominant APD and dominant spiral wave period for each model.

### Numerical methods

In all cases, we solved the following monodomain representation of cardiac tissue: 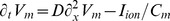, where *V_m_* is the membrane potential, *D* is the diffusion constant, *I*_ion_ is the sum of the ionic currents given by the model formulation used in each case, and *C_m_* is the membrane capacitance (set to 1 µF/cm^2^ in all cases). Both models were integrated using the explicit Euler method with uniform spatial and temporal resolutions, which facilitated parallelization in 2D. The Rush and Larsen method [24] was used to integrate the Hodgkin-Huxley-type equations of the gating variables in both models. Some variables were integrated semi-implicitly to extend the range of time steps for which the method was stable. The time step used for both the GPB and OVVR models was 0.02 ms and the spatial resolution used in all tissue simulations was 0.015 cm. For the OVVR model, we also used operator splitting to integrate the calcium concentration equations with a smaller time step of 0.001 ms, which was necessary to accurately capture the fast dynamics of these equations. To increase efficiency, pre-computed lookup tables were used to calculate single-variable computationally intensive functions, such as exponentials [25]. The diffusion coefficient used in all cases was 0.001171 cm^2^/ms, as calculated for human ventricular tissue [17]. No-flux boundary conditions were used in all tissue simulations and initial conditions were as specified in the publications of the original models [18], [19]. The length of the cables in 1D for both models was 1.5 cm and the size of the 2D tissue sheets was 14.4 cm×14.4 cm, except for the epicardial cell type of the OVVR model, where the sheet was 18.0×18.0 cm. We used the same temporal and spatial resolution for previously published models except the time resolution of the IMW model, where we used 0.01 ms. Our codes are consistent with the implementations for the models available at www.cellml.org (GPB model) and http://rudylab.wustl.edu/research/cell/code/AllCodes.html (OVVR model) with the exception that for the GPB model we set the coefficient in the equation for I_K1_ to 0.35, as in the original paper, rather than to 10.35, as in the CellML code. All other parameters are as specified in the original papers.

## Results

### Action potentials, transmembrane currents, and calcium transients

**Figure 1:**
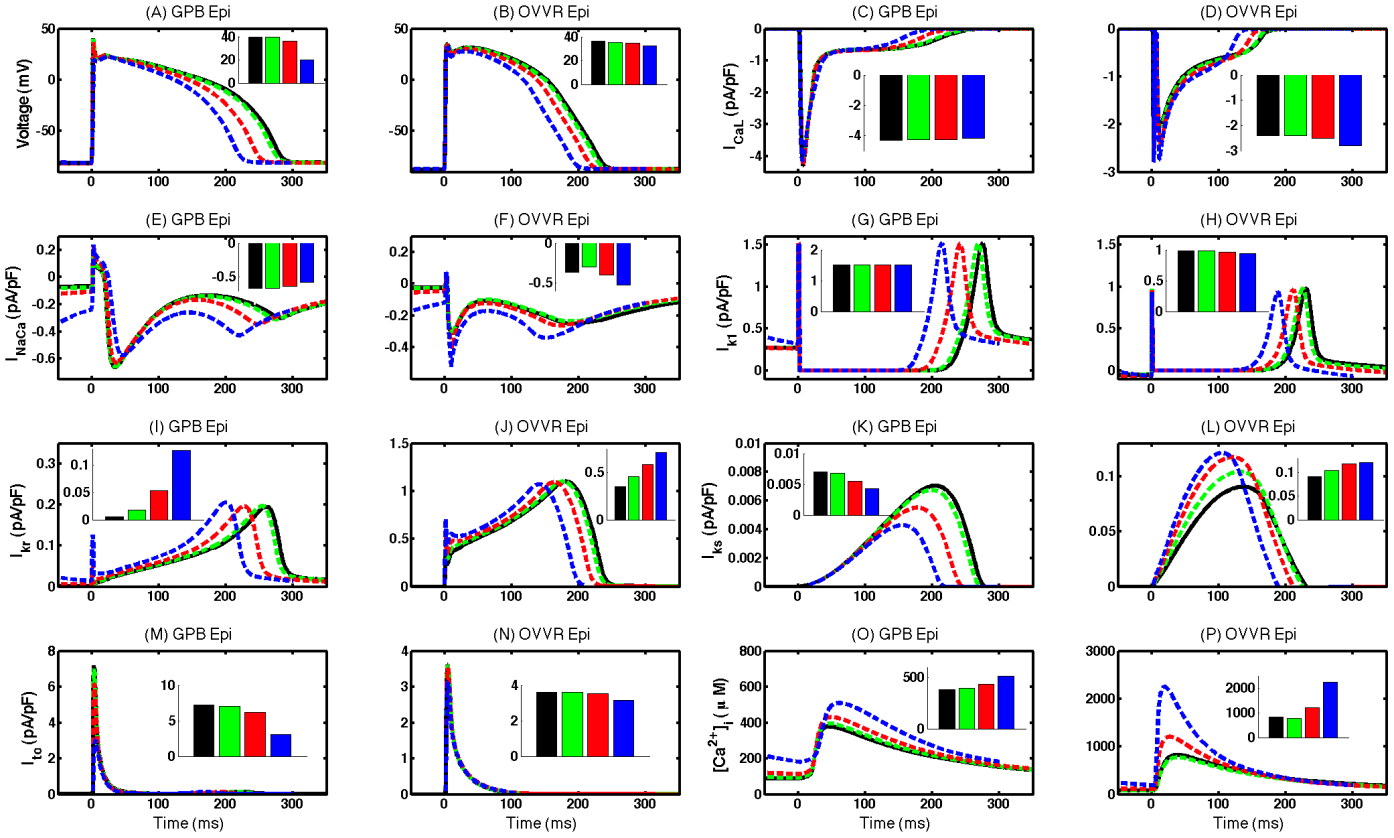
Rate dependence of action potentials, primary transmembrane currents, and intracellular calcium concentration. Action potentials, currents, and calcium transient in a single cell for the GPB (columns 1 and 3) and OVVR (columns 2 and 4) models for cycle lengths of 1000 ms (solid black), 750 ms (dashed green), 500 ms (dashed red), and 300 ms (dashed blue). Insets show peak current values for the same cycle lengths following the same color scheme. The GPB model generally shows more rate dependence; however, the OVVR model shows greater rate dependence for I_K1_ and [Ca^2+^]_i_. Both models show significant rate dependence for I_Ks_, although the effect of rate is opposite for the two models, and for the I_CaL_.

We compared the action potentials, main transmembrane currents (I_CaL_, I_NaCa_, I_K1_, I_Kr_, I_Ks_, and I_to_), and calcium transients in single cells of the GPB and OVVR models for a range of different CLs for the epicardial formulations. As shown in Fig. 1A–B, the action potentials of the two models have similar shapes, although action potentials obtained using the OVVR model have higher plateaus and shorter durations and exhibit less rate dependence than those of the GPB model.

The main transmembrane currents of the two models generally show differences in magnitude and in the degree of rate dependence. Neither model displays much rate dependence of I_CaL_ (see Fig. 1C–D), but the peak current at long cycle lengths is nearly twice as large for the GPB model (−4.3 pA/pF) as for the OVVR model (−2.4 pA/pF). I_NaCa_ exhibits stronger rate dependence for the GPB model and limited but biphasic rate dependence for the OVVR model, as shown in Fig. 1E–F. The peak inward current is twice as large for the GPB model as for the OVVR model at longer CLs (1000 ms) and is similar for the two models at short CLs (300 ms) with a value of −0.53 pA/pF, but the GPB model has a more pronounced outward component early in the action potential than the OVVR model.

I_K1_ is similar in the two models; however, the peak current is about 50% larger for the GPB model than for the OVVR model, as shown in Fig. 1G–H. In addition, I_K1_ in the GPB model displays almost no rate dependence, whereas for the OVVR model the peak value of the current decreases slightly with decreasing CL. As shown in Fig. 1I–J, I_Kr_ exhibits very slight rate dependence in both models, but in opposite directions, and its peak value for the OVVR model is six times larger than for the GPB model, indicating that it plays a more significant role during repolarization for the OVVR model. I_Ks_ also is larger for the OVVR model than for the GPB model by more than a factor of ten (see Fig. 1K–L). Both models show rate dependence of I_Ks_, but in opposite ways: as CL decreases, the peak value of I_Ks_ decreases for the GPB model but increases for the OVVR model. For I_to_, the OVVR model shows limited rate dependence, in contrast to the GPB model, where the peak value decreases considerably with decreasing CL, as shown in Fig. 1M–N. The peak current is two times larger for the GPB model than for the OVVR model at slow pacing rates, but both models have the same peak value at fast rates.

Fig. 1O–P shows the calcium transients (intracellular calcium concentration [Ca^2+^]_i_) for both models. The peak value is about twice as large for the OVVR model as for the GPB model during slow rates and more than four times as large during fast rates. In both models, the [Ca^2+^]_i_ peak value increases as the CL increases from 1000 ms to 300 ms, so that the [Ca^2+^]_i_ peak-frequency relationship is always positive. In addition, the calcium transient rises and falls more slowly for the GPB model than for the OVVR model.

### Transmural variations in action potentials

**Figure 2:**
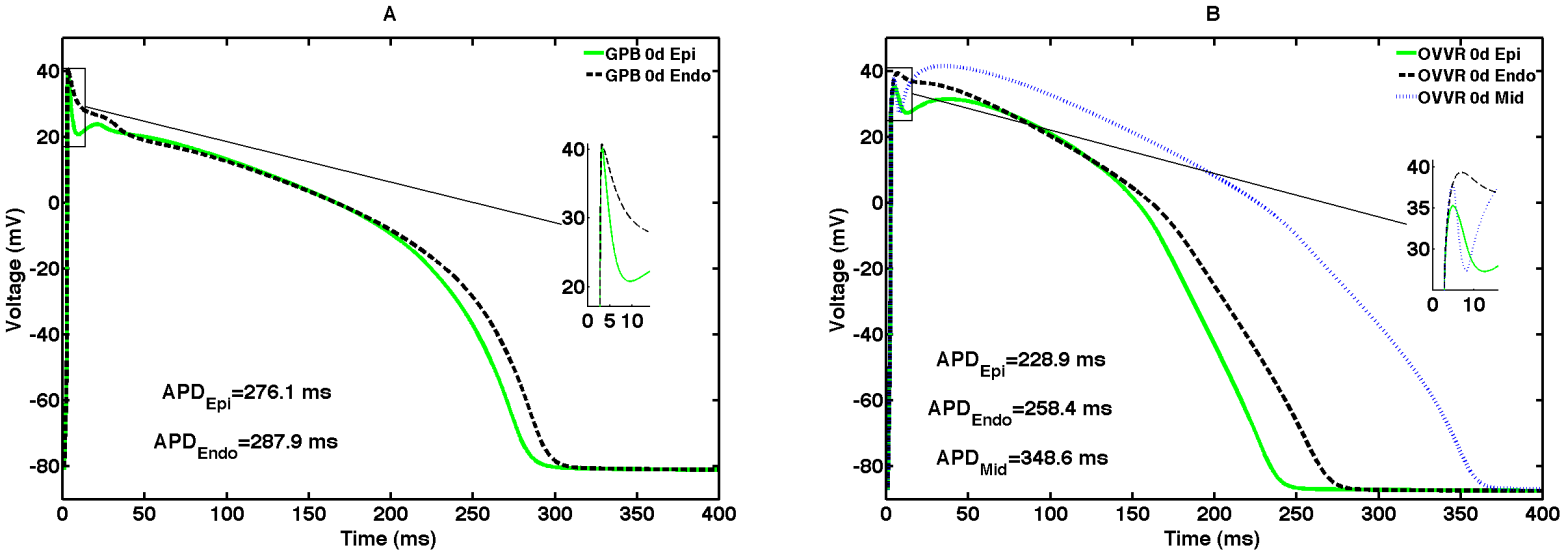
Transmural cell types. (A) Epicardial and endocardial action potentials for the GPB model. (B) Epicardial, endocardial, and midmyocardial action potentials for the OVVR model. All measurements were obtained after pacing a single cell for 30 s with a CL of 1 s.

Both the GPB and OVVR models include endocardial cell formulations, and the OVVR also includes a midmyocardial cell formulation, as shown in Fig. 2. Action potentials obtained using the endocardial formulations do not have a prominent notch because of decreased I_to_ density and are longer than epicardial action potentials. For the midmyocardial cell type, the OVVR model exhibits a prominent dome with the height of the plateau higher than the peak of the upstroke for both single cell and tissue. The APD of the midmyocardial cell for the OVVR model at a CL of 1000 ms is 348.6 ms, which is longer than that of the epicardial cell by 119.7 ms and that of the endocardial cell by 90.2 ms. The OVVR model APDs for both epicardial and endocardial cells are shorter than those of the corresponding cell types in the GPB model, and the difference in GPB epicardial and endocardial APDs is also smaller (11.8 ms), as shown in Fig. 2. Additional details are given in Table 2.

**Table 2:** Action potential characteristics for the GPB, OVVR, PB, IMW, TP, and BCF models.

Model	GPB Epi	GPB Endo	OVVR Epi	OVVR Endo	OVVR Mid	PB	IMW	TP	BCF
***Single Cell***									
**RMP (mV)**	−81.4	−81.4	−87.8	−87.9	−87.6	−90.6	−90.7	−85.4	−83.9
**Amplitude (mV)**	121.8	122.3	123.1	127.2	125.4	149.4	129.8	124.2	128.1
**V_notch_ (mV)**	20.8	-	27.3	-	27.3	4.1	19.5	16.0	6.3
**V_plateau_ (mV)**	23.9	26.9	31.6	36.5	41.6	29.1	31.9	25.2	21.9
**dV/dt_max_ (V/s)**	393.5	393.8	216.8	219.5	214.4	414.1	283.1	368.4	251.7
**APD (ms)**	276.1	287.9	228.9	258.4	348.6	393.2	324.5	304.3	264.3
**MA_APD_ (ms)**	49.5	47.0	21.9	26.2	24.0	11.0	125.6	124.7	0.0
**CL_min_ (ms)**	250	270	165	200	270	290	330	225	300
**DI_min_ (ms)**	31.5	40.4	2.1	3.3	4.9	4.8	23.7	44.7	94.0
**Maximum slope**	0.3	0.3	0.4	0.7	0.2	1.0	0.1	1.0	0.9
**Alternans onset CL**	-	-	165	280	-	-	330	220	-
***Tissue***									
**RMP (mV)**	−81.4	−81.4	−87.8	−87.9	−87.5	−90.6	−90.7	−85.4	−83.9
**Amplitude (mV)**	100.4	100.7	107.8	114.4	105.2	109.5	116.2	108.2	122.9
**V_notch_ (mV)**	-	-	-	-	-	13.6	17.7	14.3	7.0
**V_plateau_ (mV)**	23.2	26.3	32.1	35.7	41.6	29.6	31.3	25.0	22.5
**dV/dt_max_ (V/s)**	302.9	303.3	81.6	83.9	81.9	279.6	282.9	266.3	220.5
**APD (ms)**	275.0	288.0	229.7	258.6	347.8	391.9	317.4	303.4	265.7
**MA_APD_ (ms)**	56.4	56.9	22.6	28.0	23.3	11.1	43.1	16.5	0.0
**CV_max_ (cm/s)**	71.4	71.4	37.8	38.2	37.9	62.8	62.2	65.3	74.6
**MA_CV_ (cm/s)**	0.6	0.5	3.1	1.4	3.5	1.3	4.1	2.4	0.0
**CL_min_ (ms)**	260	270	320	310	400	310	330	230	290
**DI_min_ (ms)**	55.6	54.2	128.3	95.6	75.5	14.9	122.3	46.4	90.1
**Maximum slope**	0.3	0.3	0.1	0.2	0.2	0.8	0.7	0.8	1.1
**Alternans onset CL**	-	-	320	310	-	-	330	-	-
**Dominant period in 2D (ms)**	308	320	337, 481	405	430	318	121, 178, 283	233	286

Characteristics include resting membrane potential (RMP), amplitude, minimum phase 1 voltage (V_notch_), plateau voltages (V_plateau_), maximum upstroke velocity (dV/dt_max_), action potential duration (APD) at a CL of 1000 ms, APD memory amplitude (MA_APD_), minimum cycle length (CL_min_), minimum diastolic interval (DI_min_), maximum steady-state restitution curve slope, alternans onset CL, maximum conduction velocity (CV_max_), and dominant period of reentry for 2D spiral waves. Epicardial formulations are used for the TP and BCF models.

### Comparisons with other models

**Figure 3:**
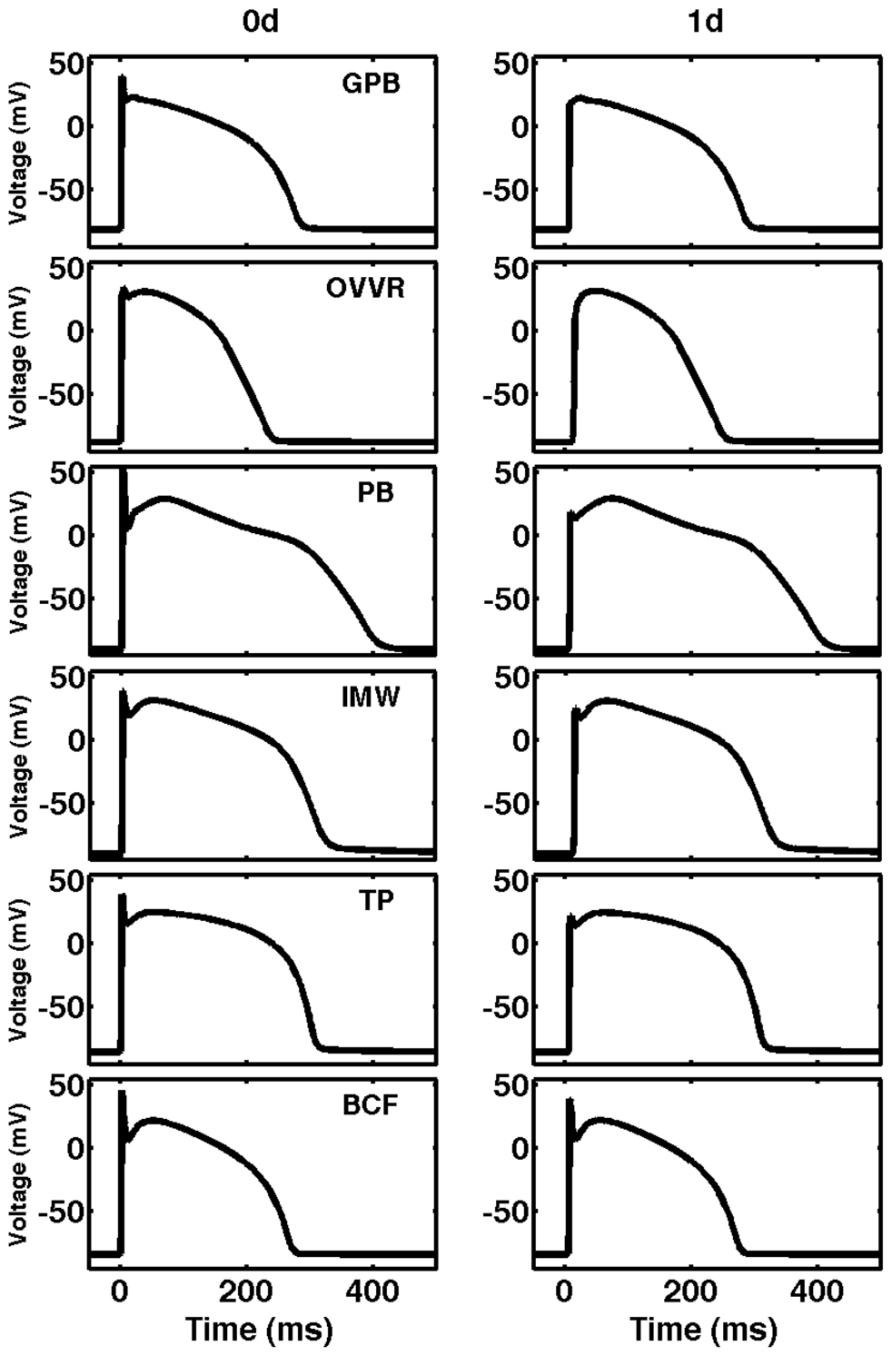
Action potentials for the epicardial formulations of six human ventricular models. Action potentials in single cells (left column) and in 1D tissue (right column). Data are taken from the middle of the cable (cell 50) with a 100 cells cable after pacing for 30 s at a CL of 1 s. Because of electrotonic coupling effects, all of the model APs lose amplitude in tissue compared to single cells, with the PB model decreasing the most (23.7%) followed by the GPB model (17.6%), the TP model (12.9%), the OVVR model (12.4%), the IMW model (10.5%), and the BCF model (4.0%).

For comparison, we also analyzed the properties of action potentials of the PB, IMW, TP, and BCF models and compared them with those of the GPB and OVVR models in single cells and in 1D cables. Figure 3 (left column) shows action potentials obtained after pacing for 30 s at a CL of 1000 ms, and Table 2 includes action potential characteristics obtained from the epicardial cell type of all four models at the same CL. In single cells, all of the models have similar spike-and-dome action potential morphologies for epicardial cells; however, the prominence of the action potential notch and the plateau potential as well as the resting membrane potential (RMP) vary among the models. The PB model has the largest upstroke peak and AP amplitude, whereas the GPB model has the smallest peak and AP amplitude. The RMP is lowest for the IMW model (−90.7 mV) and highest for the GPB model (−81.4 mV). In terms of action potential morphology, the PB model has a clear two-phase repolarization, in contrast to the other models where the transition is smoother, and the plateau phase for the TP model is longer than for the other models, for which repolarization begins sooner. APDs vary significantly among the models, ranging between 228.9 ms for the OVVR model and 393.2 ms for the PB model for epicardial cells.

In tissue, the action potential upstrokes are decreased by electrotonic effects (see Fig. 3, right column). For the OVVR model, the membrane potential continues to increase after the upstroke, so that the plateau height is greater than the upstroke depolarization, and there is no clear distinction between the upstroke and the plateau. For the GPB, PB, IMW, and TP models, a distinct upstroke spike still is observed, but with a peak lower than the plateau value. Only the BCF model has a maximum upstroke potential higher than the plateau in tissue. The maximum upstroke velocities vary considerably among the models, ranging from 81.6 V/s for the OVVR model to 302.9 V/s for the GPB model. Despite the morphological changes near the action potential upstrokes, APD does not change much between 0D and 1D; the APD in tissue increases by 0.8 and 1.4 ms for the OVVR and BCF models, respectively, and decreases by 1.1, 1.3, 7.1 and 0.9 ms for the GPB, PB, IMW, and TP models, respectively. The maximum upstroke velocity, however, decreases significantly from single cells to tissue: for the GPB model, the decrease is 23% (from 394 to 303 V/s), but for the OVVR model, the decrease is 62% (from 217 to 82 V/s for epicardial cells). The PB, TP, and BCF models also show decreases of 32%, 28%, and 12%, whereas the IMW model shows no decrease in this quantity.

### Rate dependence of APD and CV and short-term memory

**Figure 4:**
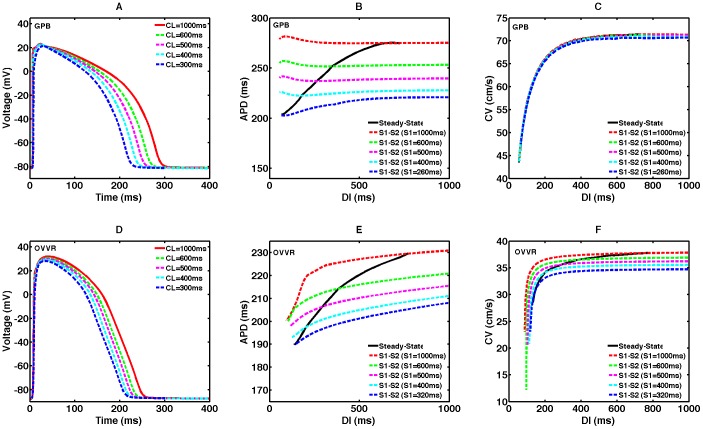
Rate dependence in a 1D cable for the GPB and OVVR models. (A,D) Action potentials at cycle lengths of 1000, 600, 500, 400, and 300 ms. Compared to isolated cell APs, the upstroke amplitude is decreased because of electrotonic effects. (B,E) Steady-state and S1–S2 APD restitution curves. Steady-state restitution curves (solid lines) were obtained after pacing for 30 s and S1–S2 restitution curves (dashed lines) were obtained after 30 s of pacing for five different S1 cycle lengths. Both models show memory in APD. (C,F) Steady-state and S1–S2 CV restitution curves. The GPB model shows no apparent memory in CV, whereas the OVVR shows limited CV memory.

Action potentials for both the GPB and OVVR models exhibit significant rate adaptation, as shown in Fig. 1AB for single cells and Fig. 4A and D for 1D cables. The steady-state restitution curves reflect this adaptation to rate, with the APD in 1D varying by 70.6 ms and 37.5 ms for the GPB and OVVR models, respectively, over CLs below 1000 ms, as shown in Fig. 4B and E (solid lines). The slopes of the steady-state restitution curves in tissue for both models are less than one over all DIs, with a maximum slope of 0.3 for the GPB model and 0.2 for the OVVR model. Similar behavior is seen for single cells (not shown).

Short-term memory, which reflects the influence of pacing history, is an important property demonstrated by both the GPB and OVVR models. The effects of short-term memory can be observed through differences in S1–S2 APD and CV restitution curves as the S1 CL is varied. Figure 4B and E (dashed lines) show S1–S2 APD restitution curves for a range of S1 CLs superimposed with the steady-state restitution curve for the GPB and OVVR models. Both models show memory; we quantify the memory using the memory amplitude, which we define as the difference between the maximum and minimum APDs over the range of S1 CLs at the longest DI of 1000 ms [6]. The memory amplitude for the epicardial cell type is considerably larger for the GPB model (54.2 ms) than for the OVVR model (22.8 ms) in a 1D cable. In terms of restitution curve shapes and slopes, S1–S2 curves for the GPB model are nearly flat, but generally become biphasic at shorter DIs (< 200 ms). In contrast, S1–S2 restitution curves decrease monotonically for the OVVR model, a phenomenon not observed for the GPB model.

**Figure 5:**
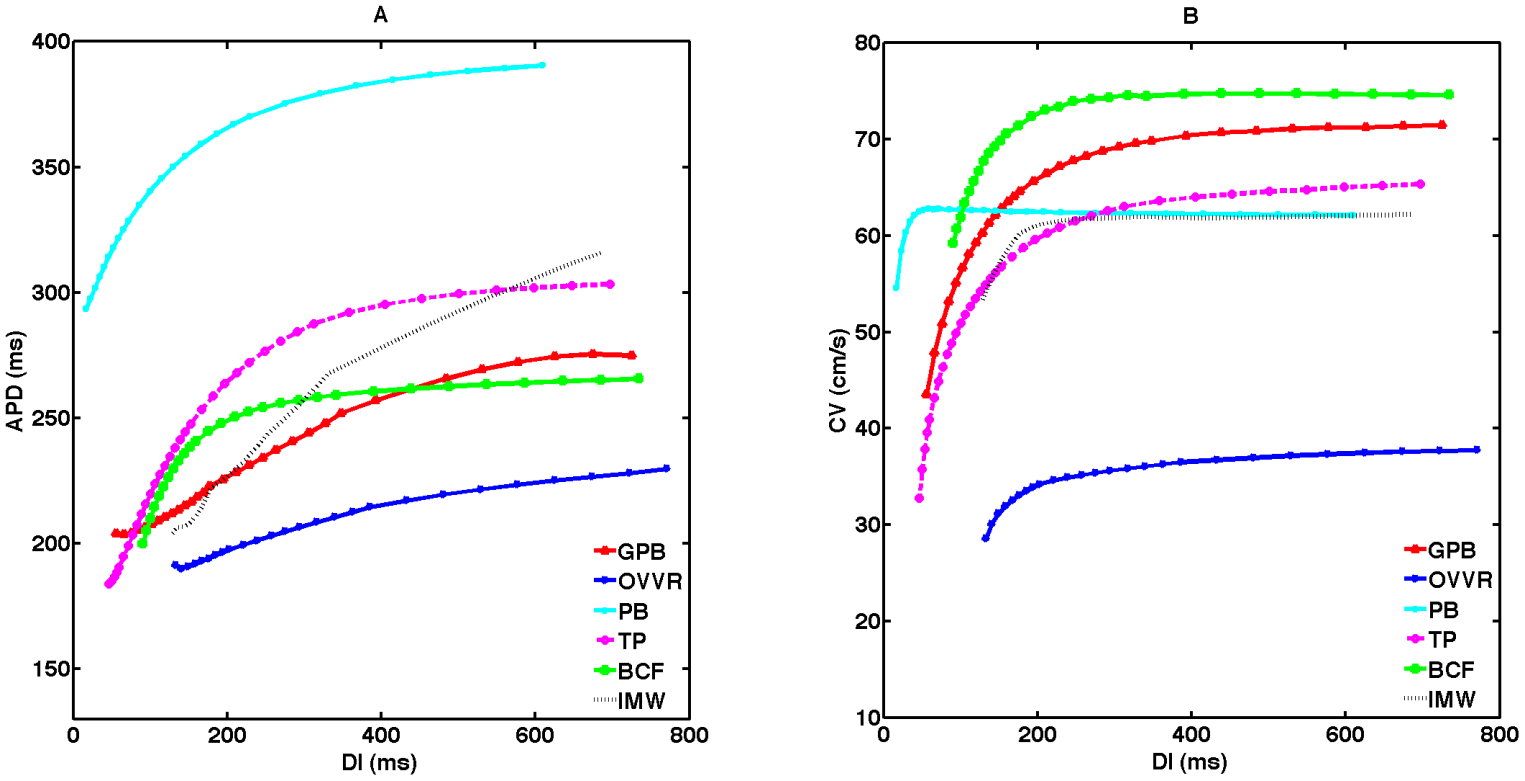
Steady-state APD and CV restitution curves for all six models in 1D epicardial cables. (A) APD restitution curves. (B) CV restitution curves. Curves were obtained after pacing for 30 s and show significant differences among the models.

Figure 5A shows steady-state APD restitution curves from all six models. All the curves decrease monotonically. However, the IMW model shows a marked decrease for long DIs and abrupt slope changes arising from lack of convergence to a steady state, which renders the model strongly sensitive to pacing protocol. For CLs below 1000 ms, APDs vary the most for the TP model (with a 119.8 ms or 39.5% decrease) and the least for the OVVR model (with a 37.5 ms or 16.4% decrease). The GPB APD range is in between, at 70.6 ms, as shown in Fig. 5A.

As shown in Fig. 4C and F (solid lines), wave propagation is considerably faster in the GPB model than in the OVVR model over all DIs; in fact, the minimum steady-state CV obtained for the GPB model in a 1D cable is larger than the maximum CV for the OVVR model. Over the range of DIs, the CV decreases by 27.9 cm/s (39.1% of the maximum CV) for the GPB model and by 9.1 cm/s (24.1% of the maximum CV) for the OVVR model.

Although all S1–S2 CV restitution curves decrease monotonically for both the GPB and OVVR models (see Fig. 4C and F, dashed lines), the GPB model exhibits almost no memory in CV; at long DIs, the CV changes by less than 1.0 cm/s as the S1 CL decreases from 1000 to 320 ms. The OVVR model shows a modest degree of memory in CV of 3.1 cm/s as the S1 CL decreases from 1000 to 165 ms.

Across all six models, the BCF model has the largest maximum CV and the OVVR model has the smallest, as shown in Fig. 5B. The CVs of the GPB and BCF models fall within a realistic range [26]–[28] with a maximum of 71.4–74.6 cm/s, and the CVs of the PB, IMW, and TP models are only slightly slower, ranging from 62.2 to 65.3 cm/s. However, the CV of the OVVR model is unphysiologically slow with a maximum of 37.8 cm/s. As discussed below, this can be remedied by substituting the TP model formulation for I_Na_. The PB model displays the least variation in CV as the DI is varied, with a steady-state CV range of 7.6 cm/s, followed by the IMW and OVVR models, with ranges of 9.0 cm/s and 9.1 cm/s, respectively. The TP and GPB models show the widest steady-state CV ranges of 32.6 cm/s and 27.9 cm/s, respectively. The steady-state CV range for the BCF model is in between, with a value of 15.3 cm/s.

### Alternans

**Figure 6:**
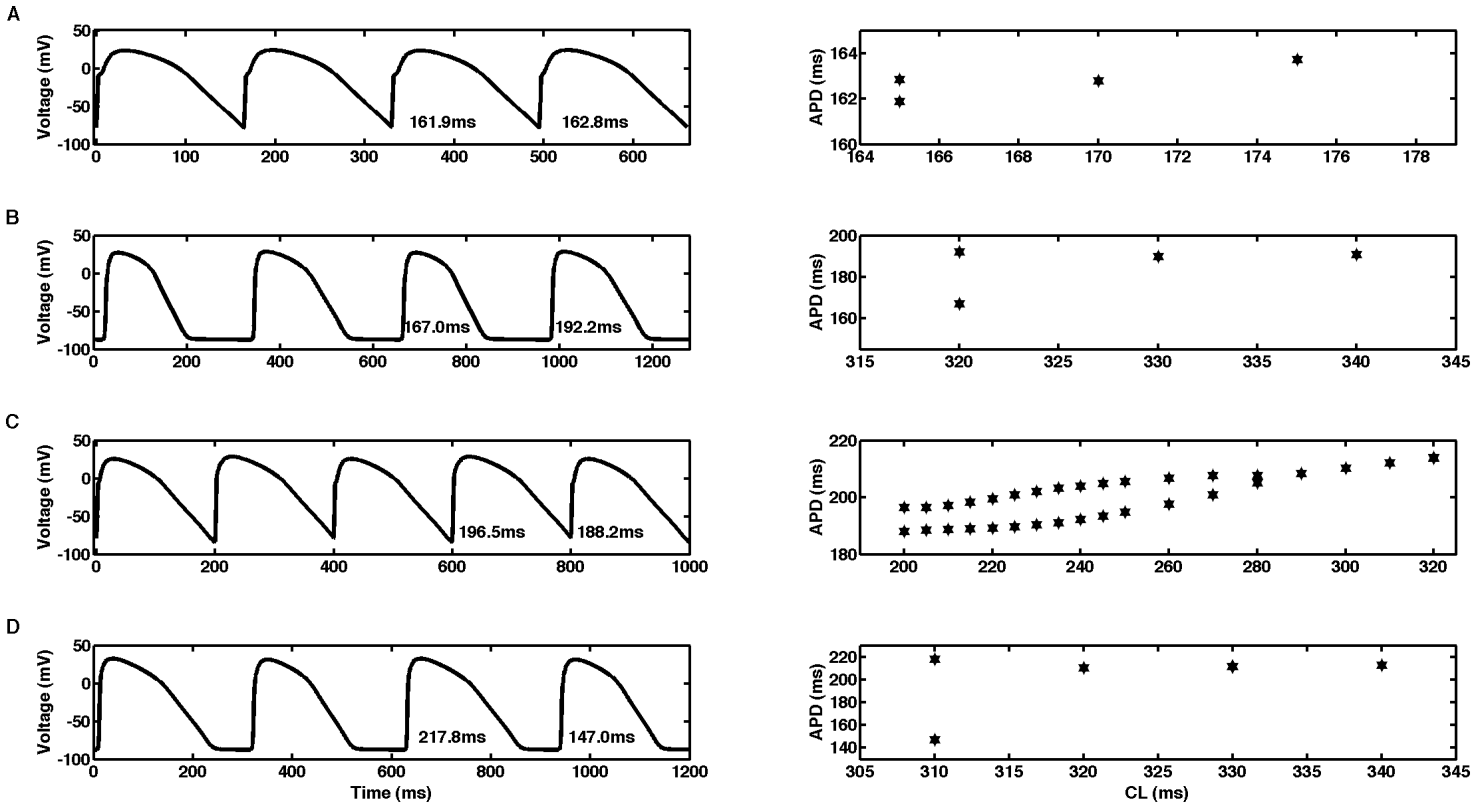
Alternans in the OVVR model. Action potential traces (left) and bifurcation diagrams (right) for (A) epicardial single cell, (B) epicardial cable, (C) endocardial single cell, and (D) endocardial cable. Cycle lengths in the action potential traces are (A) 165, (B) 320, (C) 200, and (D) 310 ms.

Alternans was never observed for the GPB model; however, it occurs for a small number of CLs in 0D and 1D for the OVVR model in both the epicardial and endocardial cell types (but not in midmyocardial cells). Fig. 6A shows that in a single epicardial cell, alternans occurs only for a CL of 165 ms, with a magnitude (difference in the APDs of two consecutive beats) of 0.9 ms. In tissue, the OVVR epicardial model shows alternans for a single CL of 320 ms with a magnitude of 25.2 ms, as shown in Fig. 6B. Alternans for a single endocardial cell occurs for CLs between 200 and 280 ms, with a maximum magnitude of 12.0 ms, as shown in Fig. 6C, but in tissue, alternans occurs only for a single CL of 310 ms with a magnitude of 70.8 ms (see Fig. 6D). Although alternans occurs in both single cells and 1D cables, the CLs exhibiting alternans were lower in 0D than in 1D for both cell types.

### Spiral wave dynamics

**Figure 7:**
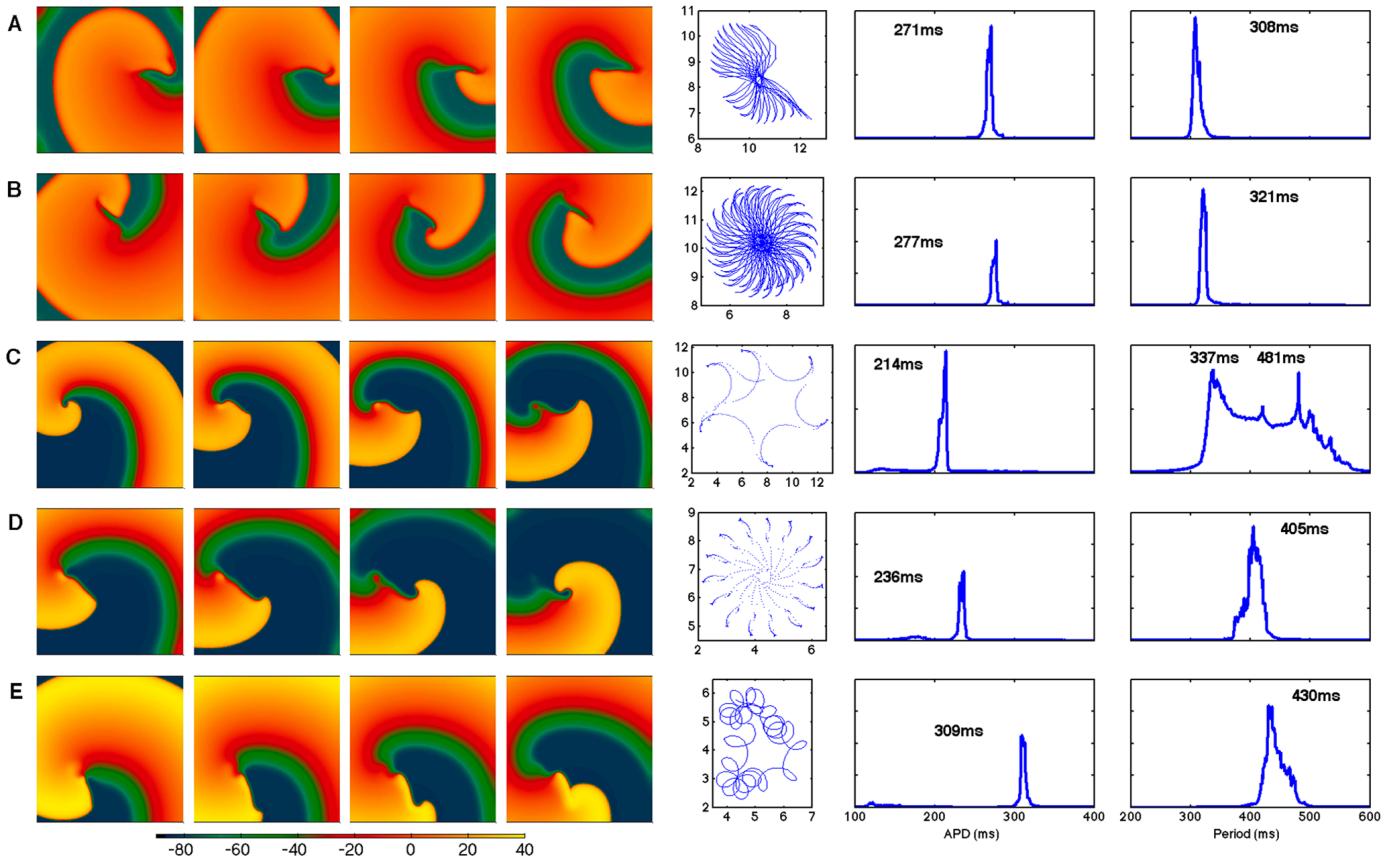
Reentrant spiral wave dynamics in 2D for the GPB and OVVR models. (A) The epicardial cell type of the GPB model features wave fronts that often stall and reform, and the dominant period is 308 ms. (B) The endocardial cell type of the GPB model shows similar stalling and recombining without breakup and a dominant period of 321 ms. (C–E) Spiral wave dynamics for (C) epicardial, (D) endocardial, and (E) midmyocardial cell types in the OVVR model. The epicardial model exhibits a quasi-breakup where a new spiral wave tip is created before the pervious one has dissipated. It has two dominant periods of 337 ms and 481 ms. The endocardial model shows similar dynamics to the epicardial formulation with a dominant period of 405 ms. The midmyocardial model features an unstable hypocycloidal trajectory with a dominant period of 430 ms. Frames in all cases correspond to 5.45, 5.50, 5.55, and 5.60 s, and tissue sizes are 14.4 cm×14.4 cm except for the OVVR epicardial cell type, where the size is 18.0×18.0 cm. Dominant periods were obtained using the full 10 s of simulation time.

The dynamics and stability of reentrant spiral waves in two-dimensional homogeneous and isotropic tissue also differ between the GPB and OVVR models, as shown in Fig. 7. Spiral waves in the GPB model feature a precessing linear tip trajectory about 4 cm long for both the epicardial and endocardial formulations (see Fig. 7AB), with the epicardial spiral wave precessing more quickly. In the OVVR model, predominantly linear trajectories are observed for the epicardial and endocardial formulations with the maximum distances traversed about 10 and 4 cm, respectively, as shown in Fig. 7CD. However, in these cases, each time the tip turns, it does so rapidly, causing it to encounter refractory tissue and die out. A new tip then forms along the spiral arm where propagation remains possible. The midmyocardial formulation shows different dynamics, with an unstable hypocycloidal trajectory featuring petals approximately 0.6 cm in length, as shown in Fig. 7E.

By recording the times between action potential upstrokes at all sites in the tissue, histograms of periods were recorded and dominant APDs and periods calculated for each case. The dominant periods for the GPB model were 308 ms and 321 ms for the epicardial and endocardial formulations, respectively. The dominant periods for the OVVR model generally were longer, with the endocardial and midmyocardial formulations showing periods of 405 ms and 430 ms, respectively. For the OVVR epicardial cell types, two prominent dominant periods of 337 ms and 481 ms were observed, with the broad spectrum of periods reflecting the especially highly meandering nature of the spiral wave for this case (note the much longer lengths in the tip trajectory compared to the other formulations).

**Figure 8:**
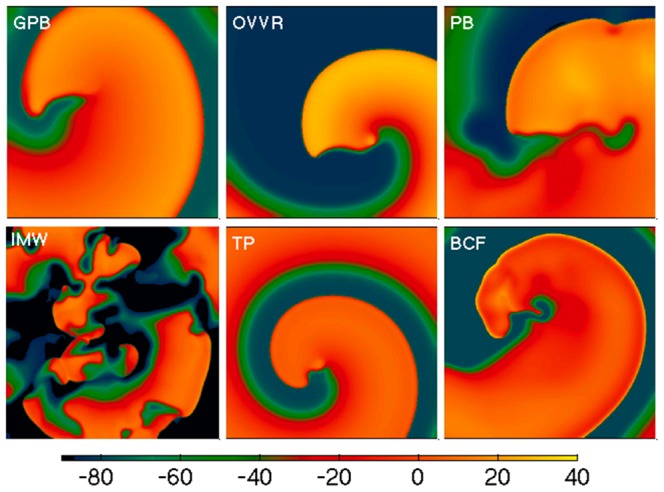
Spiral waves for all six models using epicardial formulations. Tissue sizes are 14.4×14.4 cm for the GPB model, 18.0×18.0 cm for the OVVR model, and 23.0 cm×23.0 cm for the PB, IMW TP, and BCF models. The spatial resolution is 0.015 cm in all cases and the time step is 0.02 ms except for the IMW model, where it is 0.01 ms. The IMW model used initial values corresponding to pacing a single cell at 3 Hz. Colorbar is in mV.

Figure 8 shows snapshots of spiral waves for all six models. Note that the other models exhibit a variety of dynamics, including quasi-breakup (PB model), sustained breakup (IMW model), and stable spiral waves (TP and BCF models). For the PB, TP, and BCF models, the dominant periods were 318, 233, and 286 ms, respectively. The IMW model displayed three dominant periods of 121, 178, and 283 ms and a much broader spectrum of periods overall, between about 120 and 300 ms, due to its high degree of meandering and sustained breakup.

## Discussion

### Action potential rate adaptation and APD restitution

It is important to compare the model properties with available observations, although experimental data on rate adaptation and restitution of APD in normal human tissue are somewhat limited due to the difficulty in obtaining nondiseased human cardiac tissue. Therefore, most studies are performed during other cardiac surgical procedures. Koller et al. [29] reported maximum APD restitution curve slopes of 0.97±0.16 (steady-state protocol) and 0.83±0.15 (S1–S2 protocol, S1  =  500 ms) for right ventricular endocardium. Similarly, Nash et al. [30] found a median value of 0.91 for the maximum S1–S2 restitution curve slope in human epicardium; only 27% of all electrode sites recorded slopes less than 0.5. Pak et al. [31] found even higher maximum slopes using an S1–S2 protocol in normal human tissue: 1.9±0.8 at the right ventricular outflow tract and 1.7±1.1 at the right ventricular apex, with similar values obtained for a steady-state protocol. In contrast, the maximum restitution curve slopes in the models in tissue as measured by the steady-state protocol were considerably lower: the GPB model achieved a maximum slope of 0.3 for both cells types, whereas the OVVR model maximum slopes were 0.1 for the epicardial cell type and 0.2 for both the endocardial and midmyocardial cell types. Thus, neither model achieves a maximum restitution curve slope in tissue comparable to experimentally measured values.

Bueno-Orovio et al. [32] observed APDs varying between about 165 and 205 ms for endocardial cells using the S1–S2 protocol (S1  =  500 ms). In comparison, Franz et al. [33] found a larger variation in APD, between about 190–200 ms and 245–270 ms, and they also observed biphasic S1–S2 restitution curves. In terms of the models, the GPB model has nearly flat S1–S2 APD restitution curves for nearly all S1 cycle lengths. However, the OVVR model more closely matches the experimental values, with APD varying between 197 and 215 ms. Changes to the I_Na_ formulation, as discussed below, may reduce the OVVR model minimum APD further in this case and thus achieve better agreement with the data of Bueno-Orovio et al. The OVVR model showed no indication of biphasic restitution curves (see Fig. 4E), whereas the GPB model showed an increase in APD at shorter DIs for longer S1 cycle lengths (see Fig. 4B). However, the biphasic restitution curves observed by Franz et al. showed an increase in APD at short, but not the shortest, DIs, and a significant decrease in APDs for the smallest DIs, in contrast with findings for the GPB model.

### Transmural heterogeneity

The GPB model includes epicardial and endocardial formulations, and the OVVR includes both of these as well as a midmyocardial formulation. The different cell types of the models in tissue exhibit some action potential properties similar to experimental observations, but there are also a number of differences. The amplitudes of epicardial action potentials (100.4 and 107.8 mV for the GPB and OVVR models, respectively) are smaller than experimental observations of 123 mV [34] and 131 mV [35]; most likely this results from the decreased upstroke amplitude of the model APs in tissue compared to single cells (see Fig. 3). The values of dV/dt_max_ for the epicardial formulations of the GPB and OVVR models are 302.9 and 81.6 V/s, which are significantly different from observations of 228±11 V/s [36] and 196±20 V/s [37]. The human epicardial APD at a CL of 1000 ms has been measured at 271±13 ms [35], which is nearly the same as for the GPB epicardial model (275.0 ms), but longer than that of the OVVR model (229.7 ms).

Human endocardial AP amplitudes have been measured at 119 mV [34] and 123 mV [35]; in tissue, the GPB model is still below these values with an AP amplitude of 100.7 ms, but the OVVR model amplitude of 114.4 ms is close to the experimental values. As for the epicardial models, the maximum upstroke velocities of the endocardial models are still larger (GPB model, 303.3 V/s) and smaller (OVVR model, 83.9 V/s) compared to experimental values (234±28 V/s [36] and 231±30 V/s [37]). However, the endocardial APDs for both models (288.0 ms for the GPB model and 258.6 ms for the OVVR model) are within the range of what some experimental studies have found for endocardial APD values (263±33 ms [35] and 270±7 ms [29]), although longer than reported in other studies (196.7±20.1 ms and 207.8±21.5 ms for right and left ventricular endocardium, respectively [32]).

Perhaps because of continued controversy surrounding the existence and function of midmyocardial cells [38]–[40], only the OVVR model includes a midmyocardial formulation. The model AP amplitude of 105.2 ms is lower than the experimentally observed value of 128 mV [35], and its upstroke velocity remains quite low at 81.9 V/s compared to the experimentally observed value of 326±16 V/s [36].

Overall, both the GPB and OVVR models in tissue exhibit action potential amplitudes smaller than experimental observations, the GPB model overestimates and the OVVR model underestimates the maximum upstroke velocity, and the GPB model APDs are close to experimental values for both epicardial and endocardial cells, whereas the OVVR model endocardial but not epicardial APDs are close to experimental measurements.

### Conduction velocity

Maximum conduction velocity values for the GPB model as well as for the earlier models are between 60 and 75 cm/s. This range agrees well with the range of 65–87 cm/s obtained in human heart studies [27], [28]. The minimum conduction velocity obtained has been found experimentally using an S1–S2 protocol to be 25% lower than the maximum [41], which is comparable to the OVVR model, which has a decrease of 24.1%. The GPB, however, shows a larger decrease of 39.1% using the S1–S2 protocol with an S1 CL of 1000 ms. Thus, the GPB model may show extra rate adaptation.

**Figure 9:**
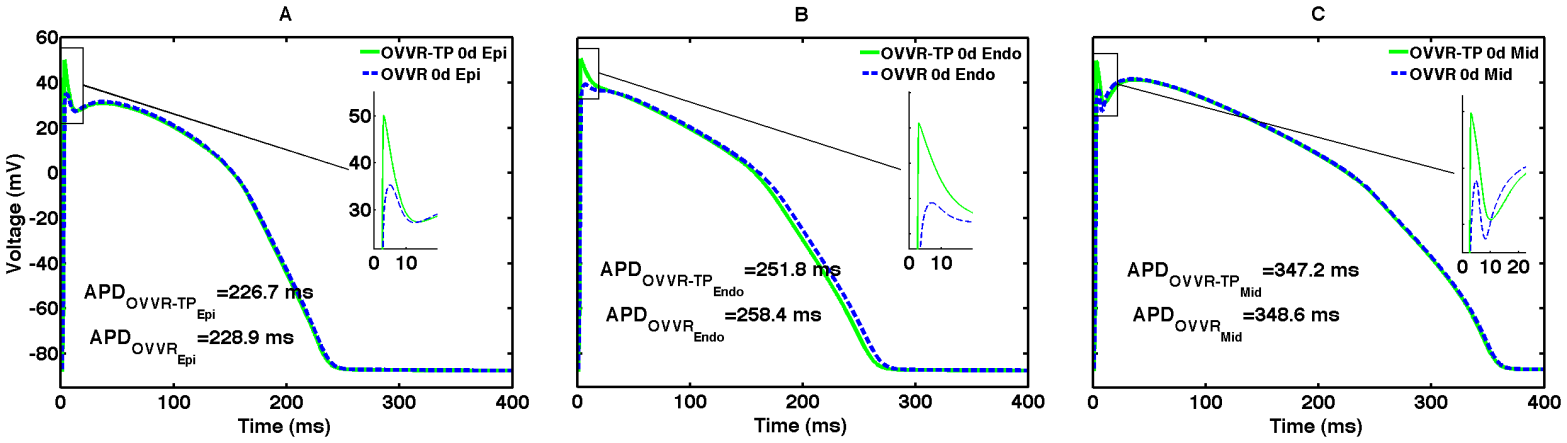
Action potentials for the OVVR model using the TP formulation of I_Na_. Traces show action potentials for (A) epicardial, (B) endocardial, and (C) midmyocardial cell types of the OVVR model using the TP model formulation of I_Na_ (green solid) compared to the original OVVR model (blue dashed). Insets show upstrokes, where the action potential shapes change most.

**Figure 10:**
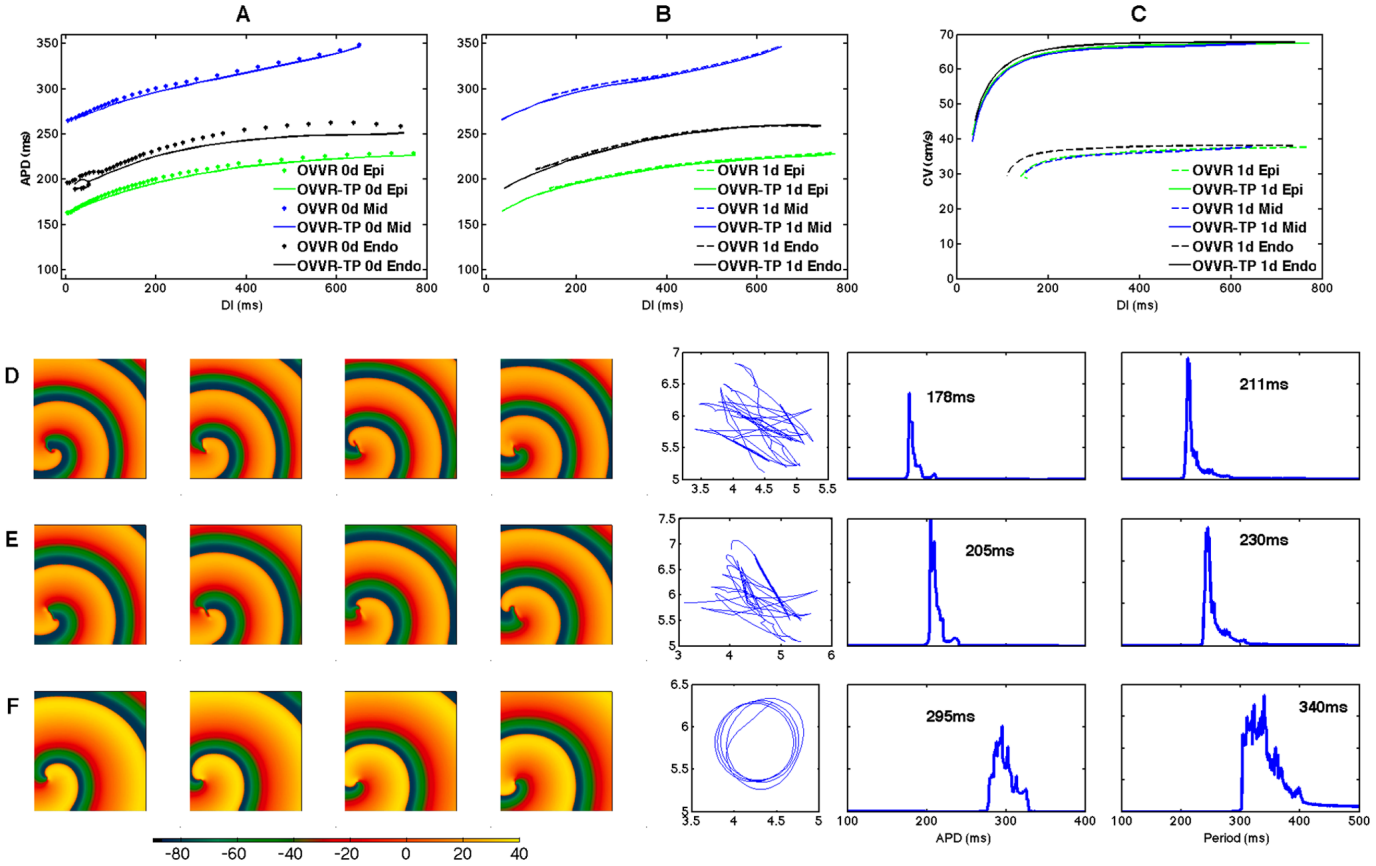
Rate adaptation and spiral wave properties for the OVVR model with the TP I_Na_ formulation. (A–B) Steady-state APD restitution curves in isolated cells (A) and a one-dimensional cable (right) for epicardial (green), endocardial (red), and midmyocardial (blue) cells. Original model restitution curves are shown as dots (A) or dashed lines (B). The different I_Na_ formulation decreases the minimum DI that can be reached in tissue. (C) Steady-state CV restitution curves for the epicardial (green), midmyocardial (blue) and endocardial (black) cells in a one-dimensional cable. Original model CV restitution curves are shown as dashed lines. The modification increases the maximum CV by almost a factor of two. (D–F) Spiral wave snapshots, tip trajectories, and dominant periods for the epicardial, endocardial, and midmyocardial formulations of the modified model. Frames in all cases correspond to 1.85, 1.90, 1.95, and 2.00 s, and tissue sizes are 18.0×18.0 cm. Dominant periods were obtained using the full 2 s of simulation time.

However, the main discrepancy where CV is concerned is in the maximum CV of the OVVR model, which, at about 38 cm/s, is about half of what has been observed experimentally. The low velocity is related to the sodium channel formulation of the OVVR model, which included temperature-related adjustments to the data of Sakakibara et al. [42] used as the basis for many I_Na_ formulations along with the novel incorporation of Ca^2+^/calmodulin-dependent protein kinase II effects. One simple remedy is to substitute the I_Na_ formulation of the TP model, which nearly doubles the maximum CV. Figure 9 shows the effect of this substitution on the epicardial, endocardial, and midmyocardial action potential shapes. In all cases, the revised sodium current increases action potential amplitude in isolated cells by 10–15 mV, but otherwise there is little effect on action potential shape; APD is decreased slightly by 2.2, 6.6, and 1.4 mV for epicardial, endocardial, and midmyocardial cells. Figure 10 demonstrates the rate adaptation and spiral wave properties of the modified model. The primary effects in tissue of the I_Na_ substitution are an increase in maximum conduction velocity to nearly twice its original value, a decrease in minimum CL by 65–115 ms (and thus the minimum APD by 20–30 ms), a decrease in dominant APD and spiral wave period, and changes to spiral wave dynamics. Spiral wave stability is not affected. Thus, the I_Na_ substitution provides a realistic CV for the OVVR model while leaving many other model properties unchanged.

### Alternans

Although alternans is known to occur in normal human hearts from both clinical [29] and ECG studies [43], the GPB model does not exhibit alternans at any CL. Alternans occurs in the OVVR model in tissue for a CL of 320 ms in epicardial cells a CL of 310 ms in endocardial cells; the magnitudes of alternans for those cell types were 25.2 and 70.8 ms, respectively. Koller et al. [29] found the alternans of onset occurred at a much lower CL of 267 ms, with a maximum alternans magnitude was 11 ms. Thus, although alternans occurs for the OVVR model but not the GPB model, alternans in the OVVR model is present earlier and achieves a significantly greater magnitude than what has been observed clinically.

### Reentrant wave dynamics

All of the GPB and OVVR model variations exhibit stable or quasi-stable dynamics, with no sustained breakup of spiral waves occurring. Well-defined periods occur in all cases except for the OVVR epicardial formulation, which has two peaks associated with the spiral wave rotation and the broadly meandering trajectory of the spiral wave. Converting these dominant periods to dominant frequencies facilitates comparison with experiments. The dominant frequencies of the GPB model, which are 3.25 and 3.12 Hz for epicardial and endocardial cells types, respectively, both lie within the range of clinically observed dominant frequencies of VT, 2.9–4.2 Hz [29] (VF frequencies are higher, up to about 7.5 Hz [28], [44], [45]). For the OVVR model, the dominant frequencies measured of 2.08, 2.47, and 2.33 Hz for epicardial, endocardial, and midmyocardial preparations, respectively, are lower than those observed clinically for VT, except for the second epicardial frequency, which at 2.97 Hz is just inside the clinical range. Thus, the GPB model corresponds well to VT, whereas the OVVR model frequencies are somewhat lower than the values typically observed for VT clinically. However, the substitution of the TP model formulation of I_Na_ changes the observed dominant frequencies of the OVVR model to 4.74, 4.35, and 2.94 Hz for the epicardial, endocardial, and midmyocardial formulations. The modification thus brings the dominant frequency for midmyocardial cells within the clinical range, with the frequencies for epicardial and endocardial cells just above that range.

Although induced reentrant waves do not produce breakup in two dimensions, it is possible that additional breakup mechanisms specific to three-dimensional tissue [46] could produce fibrillatory-like states. Further study is needed to determine how tissue thickness and anatomy affect the stability of reentry for these models.

## Conclusions

We have analyzed quantitatively the dynamics of two recently published models of human ventricular cells, the GPB model and the OVVR model, in isolated cells and in one- and two-dimensional tissue constructs and have compared the observed properties with those of other ventricular models and with available experimental and clinical data. We have shown that each model has strengths and limitations that suggest how it can be best utilized for cardiac tissue studies. The GPB model produces APDs and a maximum CV value closer to experimentally observed values along with clinically relevant dominant frequencies corresponding to VT. The OVVR model shows greater fidelity of APD variation with S1–S2 restitution curves and produces alternans, although with a magnitude greater than observed experimentally. Using the TP model formulation for I_Na_ restores the maximum CV of the OVVR model, decreases minimum DI, and increases dominant spiral wave periods. Both models exhibit action potential amplitudes and maximum restitution curve slope values below what has been reported experimentally, do not agree well with observations of maximum upstroke velocity in tissue, and show APD restitution curve maximum slopes below than typical experimental values.

Although the models studied in many cases generate different predictions, we emphasize that model disagreement may arise for many possible reasons. The models may exhibit normally observed biological variability or may reflect spatial heterogeneity other than transmural heterogeneity, such as apico-basal [30], left-right [30], [32], or other regional [30], [31] gradients. Differences also may arise from study subject differences such as age and gender. In addition, it is important to note although models generally are designed to reproduce normal cells, it is difficult to access healthy human tissue experimentally. The other models used for comparison also have limitations, although, like the GPB and OVVR models, many of them match experimental data for some properties well [17]. Thus, reproducing observed dynamical properties of the human ventricles remains a significant modeling challenge.
